# Exploration of the effect of Celastrol on protein targets in nasopharyngeal carcinoma: Network pharmacology, molecular docking and experimental evaluations

**DOI:** 10.3389/fphar.2022.996728

**Published:** 2022-11-23

**Authors:** Junjun Ling, Yu Huang, Zhen Sun, Xiaopeng Guo, Aoshuang Chang, Jigang Pan, Xianlu Zhuo

**Affiliations:** ^1^ Affiliated Hospital of Guizhou Medical University, Guiyang, Guizhou, China; ^2^ Cancer Hospital of Guizhou Medical University, Guiyang, Guizhou, China; ^3^ School of Basic Medicine, Guizhou Medical University, Guiyang, Guizhou, China

**Keywords:** Celastrol, nasopharyngeal carcinoma, drug targets, network pharmacolgy, traditional Chinese medicine

## Abstract

**Background:** Celastrol, an important extract of *Tripterygium wilfordii*, shows strong antitumor activity in a variety of tumors including nasopharyngeal carcinoma (NPC). However, little is known about its targets in NPC. We aimed to screen the key gene targets of Celastrol in the treatment of NPC by means of *in silico* analyses (including network pharmacology and molecular docking) and experimental evaluations.

**Methods:** The main target genes of Celastrol and the genes related to NPC were obtained by retrieving the relevant biological databases, and the common targets were screened. Protein-protein interaction analysis was used to screen the hub genes. Then, a “compound-target-disease” network model was created and molecular docking was used to predict the binding of Celastrol to the candidate hub proteins. Afterward, the expression changes of the candidate genes under the administration of Celastrol were verified *in vitro* and *in vivo*.

**Results:** Sixty genes common to Celastrol and NPC were screened out, which may be related to numerous biological processes such as cell proliferation, apoptosis, and tube development, and enriched in various pathways such as PI3K- Akt, EGFR tyrosine kinase inhibitor resistance, and Apoptosis. The tight binding ability of the candidate hub proteins (TNF, VEGFA, and IL6) to Celastrol was predicted by molecular docking [Docking energy: TNF, −6.08; VEGFA,−6.76; IL6,−6.91(kcal/mol)]. *In vitro* experiments showed that the expression of TNF and VEGFA decreased while the expression of IL6 increased in NPC cells (CNE2 and HONE1) treated with Celastrol. *In vivo* experiments suggested that Celastrol significantly reduced the weight and volume of the transplanted tumors in tumor-bearing mice *in vivo*. The expression of TNF, VEGFA, and IL6 in the transplanted tumor cells could be regulated by using Celastrol, and the expression trends were consistent with the *in vitro* model.

**Conclusion:** Several gene targets have been filtered out as the core targets of Celastrol in the treatment of NPC, which might be involved in a variety of signaling pathways. Hence, Celastrol may exert its anti-NPC activity through multiple targets and multiple pathways, which will provide new clues for further research. Future experiments are warranted to validate the findings.

## Introduction

Nasopharyngeal carcinoma (NPC) is a kind of malignant tumor that mainly occurs in nasopharyngeal epithelial cells. It is common in Southeast Asian countries and in Southeast China (Su et al., 2022). The onset is concealed, and the main pathological types of most cancers are medium and low-differentiated squamous cell carcinoma. Clinically, radiotherapy combined with chemotherapy has achieved favorable results in early-stage patients, but a considerable number of patients still experience metastasis and recurrence, thus affecting their prognosis (Peng et al., 2021). Therefore, other prevention and treatment strategies need to be developed in the clinic.

In recent years, more attention has been focused on the efficacy of natural bioactive products for NPC treatment, which not only have strong biological activity but also are safe and lack systemic toxic effects (Huang et al., 2021). For example, Curcumin is a polyphenolic natural product, which can sensitize NPC cells to radiation through modulation of ROS generation, Jab1/CSN5, and non-coding RNAs (Momtazi-Borojeni et al., 2018). Asiatic acid, extracted from Centella Asiatica, was found to have anticancer activity in various cancers, including NPC. It significantly reduces the cell viability of cisplatin-resistant NPC cell lines caused by apoptosis through both the intrinsic and extrinsic apoptotic pathways (Liu et al., 2020). Apigenin is a naturally occurring plant flavone. A combination of Apigenin and Cetuximab (a well-known epidermal growth factor receptor inhibitor) could effectively suppress the viability of NPC cells by increasing G2/M phase arrest (Hu et al., 2018). Thus, the active components in natural plants may become potential anticancer drugs.

Celastrol, an important active substance in *Tripterygium wilfordii* (*T. wilfordii*), has strong anticancer activity. Celastrol is rich in the roots, stems, and leaves of *T. wilfordii*, and it has three terpenes in its structure (Jin et al., 2022). Evidence showed that Celastrol has a wide range of pharmacological effects, such as anti-inflammation, immunosuppression, anti-obesity, and anti-tumor activity. Among these biological activities, anti-tumor activity has attracted much attention (Bai et al., 2021). Celastrol exerts an inhibitory effect on breast cancer, prostate cancer, lung cancer, colorectal cancer, osteosarcoma, gastric cancer, and liver cancer (Lim et al., 2021). Moreover, it also exhibits a significant inhibitory effect on NPC cells. For instance, Celastrol induces apoptosis through the death receptor and the mitochondrial pathway in NPC cells possibly through activation of MAPK 8/9 and inhibition of (MAPK) 1/3 pathways (Lin et al., 2017). Likewise, through the modulation of MAPK pathways, Celastrol might trigger the apoptosis of cisplatin-resistant NPC cells (Hsieh et al., 2019). This suggests that Celastrol may exert anti-tumor activity by regulating the expression of some genes. However, limited studies have focused on this issue, which needs to be further explored.

**FIGURE 1:**
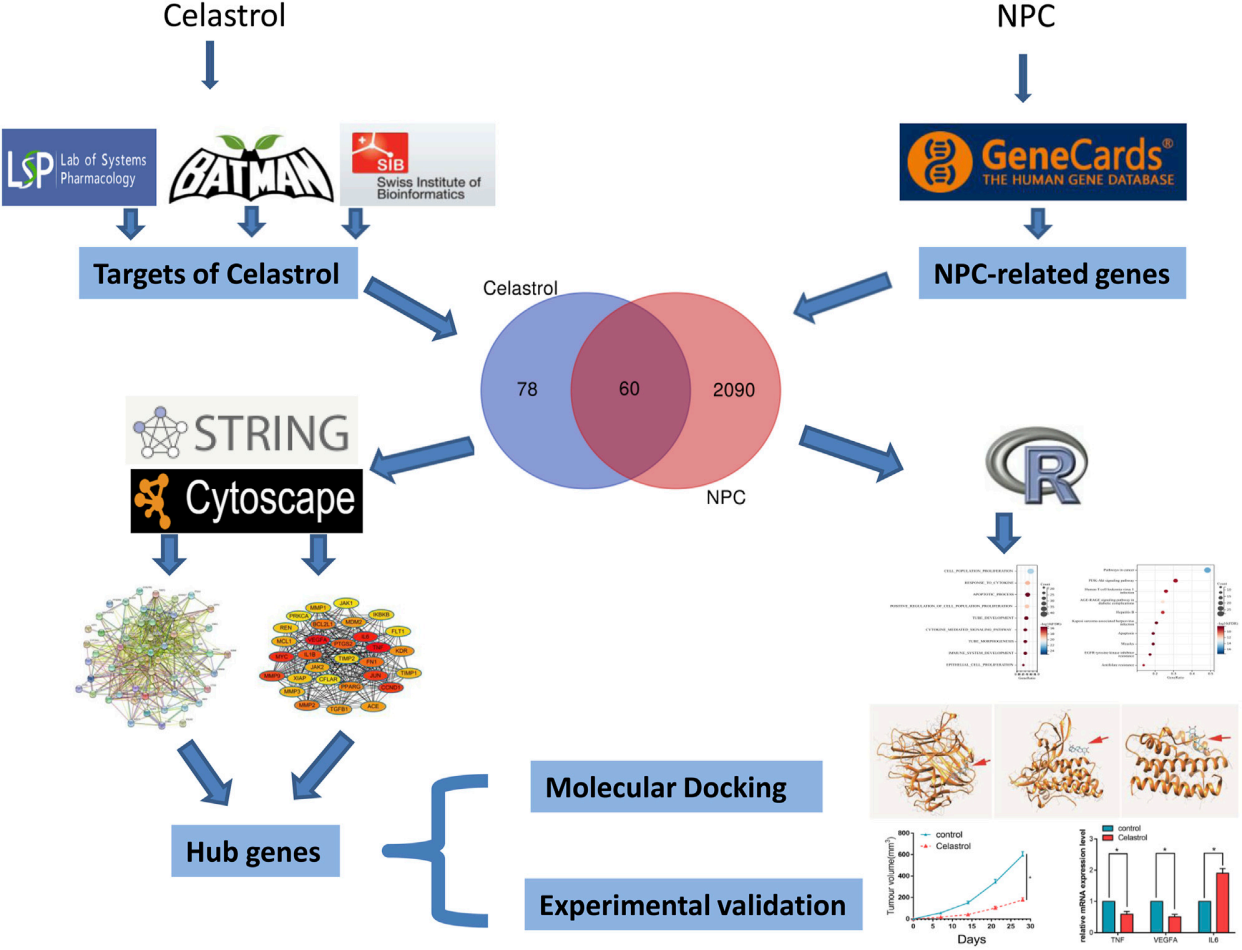
The flowchart of this study. The target genes of Celastrol were firstly obtained by screening the TCMSP, BATMAN-TCM, and Swisstarget databases; then NPC-related genes were obtained by the Genecards database; the two gene sets were intersected to obtain 60 genes. These genes were then subjected to functional enrichment analysis and PPI network construction to filter the hub genes, and the candidate hub genes were then subjected to molecular docking and experimental validation.

Traditional research often has some limitations because it only focuses on a single target or pathway. With the development of biological big data, the derived bioinformatics methods can overcome this difficulty. Network pharmacology is a branch of bioinformatics, which integrates biology, bioinformatics, pharmacology, and other disciplines, from looking for a single target gene to exploring a comprehensive network (Noor et al., 2022). It also explores the effects and possible mechanisms of drugs and target genes from multiple angles, levels, and stages. To explore the possible molecular mechanism of Celastrol in the treatment of NPC, we used bioinformatics methods (including network pharmacology) to comprehensively explore the target genes. Then, further *in vitro* and *in vivo* experiments were conducted to validate the expression of the key genes (Figure 1). The mRNA and protein expression of the key genes were detected by RT-PCR and western blot assays.

## Materials and methods

### Analysis of the pharmacokinetic parameters of Celastrol

TCMSP analysis platform is a public pharmacological analysis platform for the investigation of natural substances and traditional Chinese medicine (Ru et al., 2014). The pharmacokinetic parameters of Celastrol were obtained through the TCMSP database.

### Celastrol target screening

In addition to the TCMSP, we also used the BATMAN-TCM (Liu et al., 2016), Swisstarget platform (www.swisstargetprediction.ch), TCMIP (Wang et al., 2021), and other platforms for target screening.

First, we obtained the target gene of Celastrol by using the TCMSP platform. Second, the smile structure of Celastrol was introduced into the Swisstarget platform, and the probability (probability >0) was set as the screening condition to obtain the predicted target genes of Celastrol. Third, the target genes with scores >20 were screened using the BATMAN-TCM tool.

After obtaining the target gene/protein name, the protein name was corrected using the UniProt database (www.uniprot.org) to obtain the target gene name. Finally, the target genes obtained by the above two approaches were combined to obtain the predicted targets gene of Celastrol.

### Screening of the genes related to nasopharyngeal carcinoma

To obtain the genes related to NPC, we searched the genes related to NPC through the Genecards tool (www.genecards.org) (Stelzer et al., 2011). The keyword “nasopharyngeal carcinoma” was used to obtain the gene targets related to the disease.

### Screening of the common gene targets related to Celastrol and nasopharyngeal carcinoma

To obtain the common target genes of Celastrol and NPC, we analyzed the intersection of Celastrol target genes obtained in the above steps and NPC-related genes. The common related targets were obtained based on the Venn diagram. These genes can be considered potential target genes of Celastrol on NPC.

### Construction of the common target protein interaction network

Interactions may be observed between proteins. We used the STRING database (www.string-db.org) (Mei, 2018) to predict and analyze protein interactions. The common targets of Celastrol and NPC were used as input into the tool, and “*Homo sapiens”* was selected to construct the protein-protein interaction network (PPI network).

The cytohubba plug-in of the Cytoscape tool was used to calculate the gene network and sorted according to the degree value, and the hub genes were screened.

### Biological function annotation and pathway enrichment analysis of common targets

To understand the biological functions and the possible enriched signaling pathways of the common target genes, we performed Gene Ontology (GO) and Kyoto Encyclopedia of Genes and Genomes (KEGG) pathway enrichment analysis to learn the possible molecular mechanism of Celastrol in the treatment of NPC.

For GO analysis, the c5.go.bp.v7.4.symbols.gmt subset from the Molecular Signatures Database was used as the background, while for KEGG analysis, the KEGG rest API was used to obtain the latest gene annotation of the KEGG pathway as the background. The genes were mapped to the backgrounds. Then, the R software package clusterprofiler (version 3.14.3) for enrichment analysis was utilized for analysis. The false discovery rate (FDR) < 0.05 was considered statistically significant.

### Establishment of “compound-target-disease” network

Cytoscape 3.2.0 software was used to construct the “compound-target-disease” network interaction model, in which the “node” represents the compound, disease, or target and the “edge” represents the interaction relationship.

### Molecular docking prediction

To verify the possible binding force between Celastrol and the hub proteins, we used molecular docking technology.

First, the mol2 format of the compound directly was obtained by the TCMSP tool. Then, the crystal structures of candidate proteins were obtained from the RCSB PDB database (www.rcsb.org) (Kouranov et al., 2006), and the ligand and water molecules were removed using the PyMOL software. Next, molecular docking was carried out by using the Swissdock tool (Bitencourt-Ferreira and de Azevedo, 2019), and the results were imported into USCF chimera 1.14 software for visualization and analysis.

According to the estimated Δ*G* (kcal/mol) value, the binding force between the drug and protein was determined. A negative value indicates that they can be combined. If the negative value is below −7, the drug and protein can be very tightly coupled.

### Validation of the key targets through *in vitro* assays

The human NPC cell line, the CNE2, and HONE1 cells were conserved in our Laboratory. The use of the cell lines was approved by our institutional research ethics committee. The cells were cultured in Dulbecco’s Modified Eagle’s Medium (DMEM) containing 5% fetal bovine serum in a humidified atmosphere with 5% CO_2_ at 37 °C. The cells were respectively separated into two groups: the Celastrol-treated group (10 μM Celastrol) and the control group (without Celastrol) (Yao et al., 2019).

After intervention for 24 h, the mRNAs of the top hub genes (TNF, VEGFA, IL6) were detected by RT-PCR assay as described previously (Ling et al., 2022). Each sample was repeated in triplicate. GAPDH was used as the internal reference. The Primer sequences were listed as follows and all listed primers were 5′–3′.

TNF (F: ACG​GCA​TGG​ATC​TCA​AAG​AC, R: AGA​TAG​CAA​ATC​GGC​TGA​CG); VEGFA (F: CAA​GAC​AAG​AAA​ATC​CCT​GTG​G, R: GCT​TGT​CAC​ATC​TGC​AAG​TAC​G); IL-6 (F: TGT​GCA​ATG​GCA​ATT​CTG​AT, R: GGT​ACT​CCA​GAA​GAC​CAG​AGG​A); GAPDH (F: ATT​CCA​CCC​ATG​GCA​AAT​TC, R: GCA​TCG​CCC​CAC​TTG​ATT)

The protein expression of the three top genes was detected by western blot assay as previously described (Ling et al., 2022). In brief, cells were harvested, washed with ice-cold PBS, and lysed with RIPA buffer supplemented with protease inhibitors. Proteins were running on a 10% SDS–polyacrylamide gel electrophoresis and transferred to polyvinylidene difluoride membranes (Roche). Blots were then incubated in fresh blocking solution with an appropriate dilution of primary antibody at 4°C for 24 h. Then, the appropriate peroxidase-conjugated secondary antibody was added, incubated for 2 h, and washed with TBST. Each experiment was conducted in triplicate.

The sources of antibodies were as follows: Tumor necrosis factor alpha (TNF/TNFa) mouse Monoclonal, Vascular endothelial growth factor A (VEGFA) rabbit polyclonal, Interleukin-6 (IL6) rabbit polyclonal, and GAPDH rabbit polyclonal (BIOSS, China). The bands were visualized and quantified using the Image-Pro Plus 6.0 software (Media Cybernetics, United States).

Cell viability was evaluated by Cell counting kit-8 assay (CCK-8; ImmunoWay Biotechnology Company Plano, TX, United States). Cells were reseeded into a 96-well culture plate at a density of 4×10^3^ in the logarithmic growth phase. After a specific length of time after each group of cells had been cultured, CCK-8 reagent was added to the plates and OD450 values were monitored.

### Validation of the key targets through *in vivo* assays

To learn the efficacy of Celastrol on the expression of the candidate genes, animal experiments were further carried out. All animal experimental protocols were in accordance with the principles established by the Animal Care and Use Committee of our institute. Female BALB/c nude mice between 4 and 5 weeks of age were purchased from Charles River Laboratories (Beijing, China).

From February to April 2022, the observation was performed. For the nude mice xenograft model, HONE1 cells were subcutaneously injected. Twelve mice were divided randomly into two groups (the Celastrol-treated and the control). Then, 2 mg/kg Celestrol (dissolved in dimethyl sulfoxide and diluted in saline to final concentration) or saline (equal volumes) was intraperitoneally administered every 2 days in each group (Cai et al., 2022). The tumor volume was measured every 7 days after the tumor was measurable, and the tumor volume was measured and calculated by the formula (length×width2)/2 . On day 28, all mice were euthanized, and the tumors were excised and weighed. Next, the mRNA expression of the candidate genes was detected in the transplanted tumors by RT-PCR assay.

### Statistical analysis

Differences between groups were assessed with a *t*-test, Analysis of Variances, or a Wilcoxon Rank Sum Test according to the concrete types of the data. These analyses were performed by using the MedCalc software (15.2.2; Mariakerke, Belgium). *p* < 0.05 was considered statistically significant.

## Results

### Pharmacokinetic parameters of Celastrol

**FIGURE 2:**
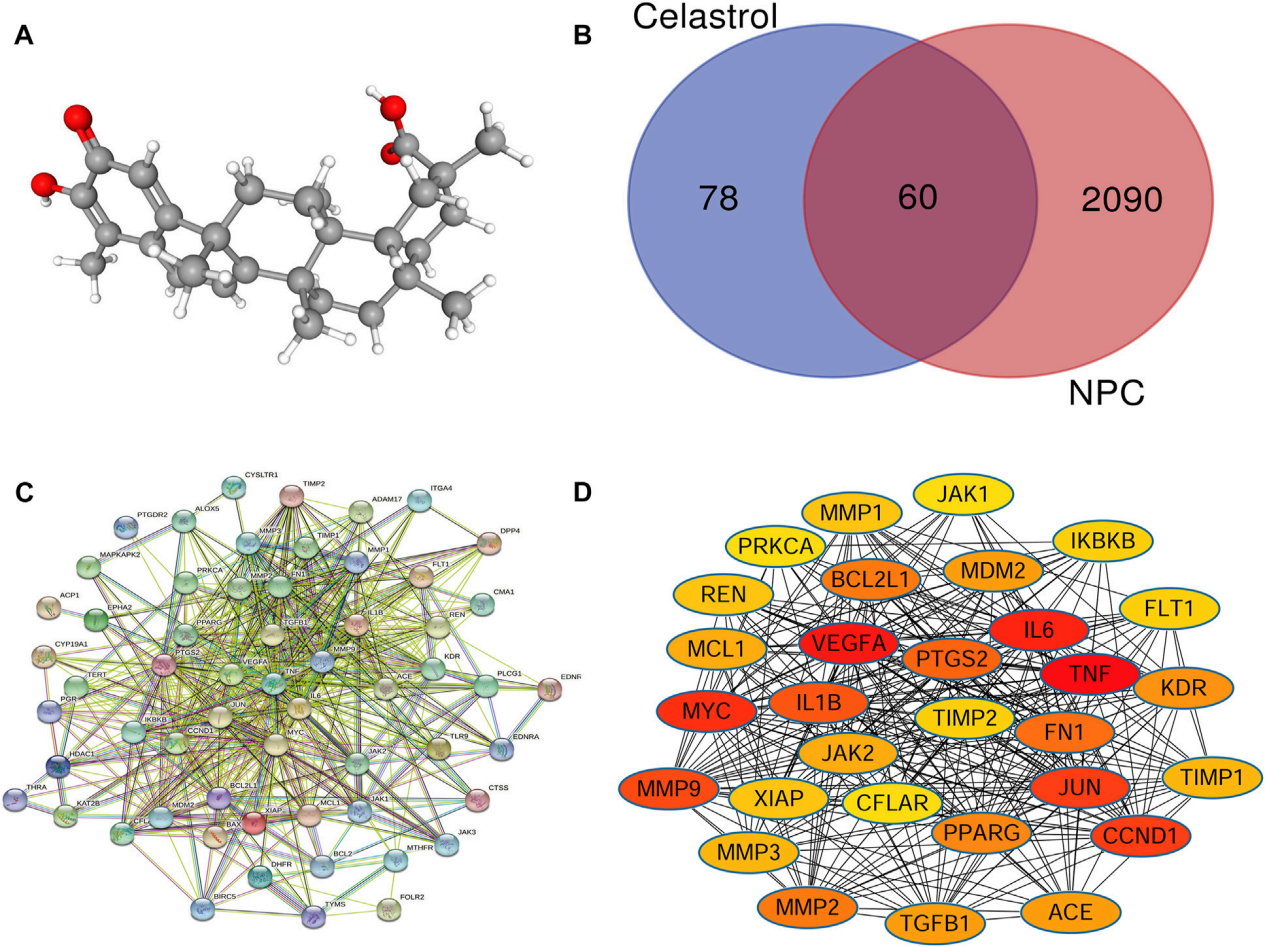
**(A)** The 3D structure of Celastrol. **(B)** Venn analysis showed that the intersection involved the genes shared by NPC and Celastrol. There were 138 targets of Celastrol and 2150 NPC-related genes. The intersection included 60 genes common to both NPC and Celastrol. **(C)** The PPI network of the common genes constructed in the STRING. **(D)** The PPI network of the top 30 genes created by Cytoscape (sorted by the “degree” values). The node represents the genes/proteins and the edges stand for the relationships.

From the TCMSP database, we obtained the pharmacokinetic parameters of Celastrol. Its molecular ID is mol003186, its molecular name is tripterine, its molecular formula is C_29_H_38_O_4_, and its relative molecular weight is 450.6 (Figure 2A). The ratio of lipid water partition coefficient (ALOGP) was 5.28, the intestinal epithelial permeability (Caco-2) was 0.46, the oral bioavailability (OB%) was 17.84, the blood−brain barrier was 0.34, and the drug-like (DL) was 0.78.

ALOGP reflects the lipophilicity of the compound (Peng and Shahidi, 2022). The intestinal permeability reflects the difficulty of drug absorption through the intestinal tract *in vivo*, which directly affects the OB of drugs (Khalid Danish et al., 2022). The blood-brain barrier represents the ability of a drug to penetrate the blood-brain barrier and is mainly demonstrated by the ratio of its concentration in the brain to that in the blood, whose value is influenced by factors such as octanol-water partition coefficient (Zhu et al., 2018). If the OB value is less than 30%, it is considered as low; if the DL value was more than 0.18, it is regarded as high (Lin et al., 2022). Based on the combined analysis of the above parameters, Celastrol has a high probability of becoming a drug, but relatively poorer water solubility and poorer oral bioavailability.

### Prediction and analysis of the common targets of Celastrol and nasopharyngeal carcinoma

In order to get the targets of Celastrol, a search in the TCMIP, TCMSP, BATMAN-TCM, and Swisstarget databases was conducted. As a result, the target genes could be obtained in the TCMSP, BATMAN-TCM, and Swisstarget tools. Nevertheless, no targets were obtained in the TCMIP database. A total of 27 targets were obtained from the TCMSP database, 100 targets from the Swisstarget database, and 18 targets from the BATMAN-TCM. As a result, a total of 145 target genes were primarily obtained. After the three sets of targets were combined and the duplicated genes were removed, 138 target genes were lastly obtained.

To get the NPC-related genes, a search in the Genecards database was performed. As a consequence, 2,150 genes related to NPC were obtained.

The intersection of the two target gene sets above was obtained based on the Venn diagram, which contained 60 possible target genes of Celastrol for NPC treatment (Figure 2B **)**.

### Protein-protein interaction network of the common target genes of Celastrol and nasopharyngeal carcinoma

To obtain the pivotal genes that play an important role in the biological process, the common targets obtained in the above process were imported to the STRING tool for protein-protein interaction network construction.

The network contains 59 nodes and 1,050 edges. Each node represents a gene/protein. Each edge represents the interaction between the proteins. The more edges represent the closer the interaction, as shown in Figure 2C.

To determine the key genes in the network, we used the Cytoscape software to calculate the degree value and sort them according to the value. The top 10 genes were ranked according to the degree value in Table 1. These genes were in pivotal positions in the network, such as TNF, VEGFA, IL6, Myc, and CCND1 (Figure 2D). Among these, the top three hub genes were chosen for further validation.

**TABLE 1:** The hub genes from the PPI network.

Node	Description	Degree
TNF	Tumor necrosis factor	100
VEGFA	Vascular endothelial growth factor A	88
IL6	Interleukin-6	84
MYC	Myc proto-oncogene protein	78
CCND1	G1/S-specific cyclin-D1	76
JUN	Transcription factor AP-1	76
MMP9	Matrix metalloproteinase-9	74
IL1B	Interleukin-1 beta	72
PTGS2	Prostaglandin G/H synthase 2	68
FN1	Fibronectin 1	64

### Gene ontology function enrichment and kyoto encyclopedia of genes and genomes pathway enrichment analysis

GO enrichment analysis mainly includes “biological process” (BP), “cellular component”, and “molecular function”. In the present study, we selected the most commonly used BP for analysis.

**FIGURE 3:**
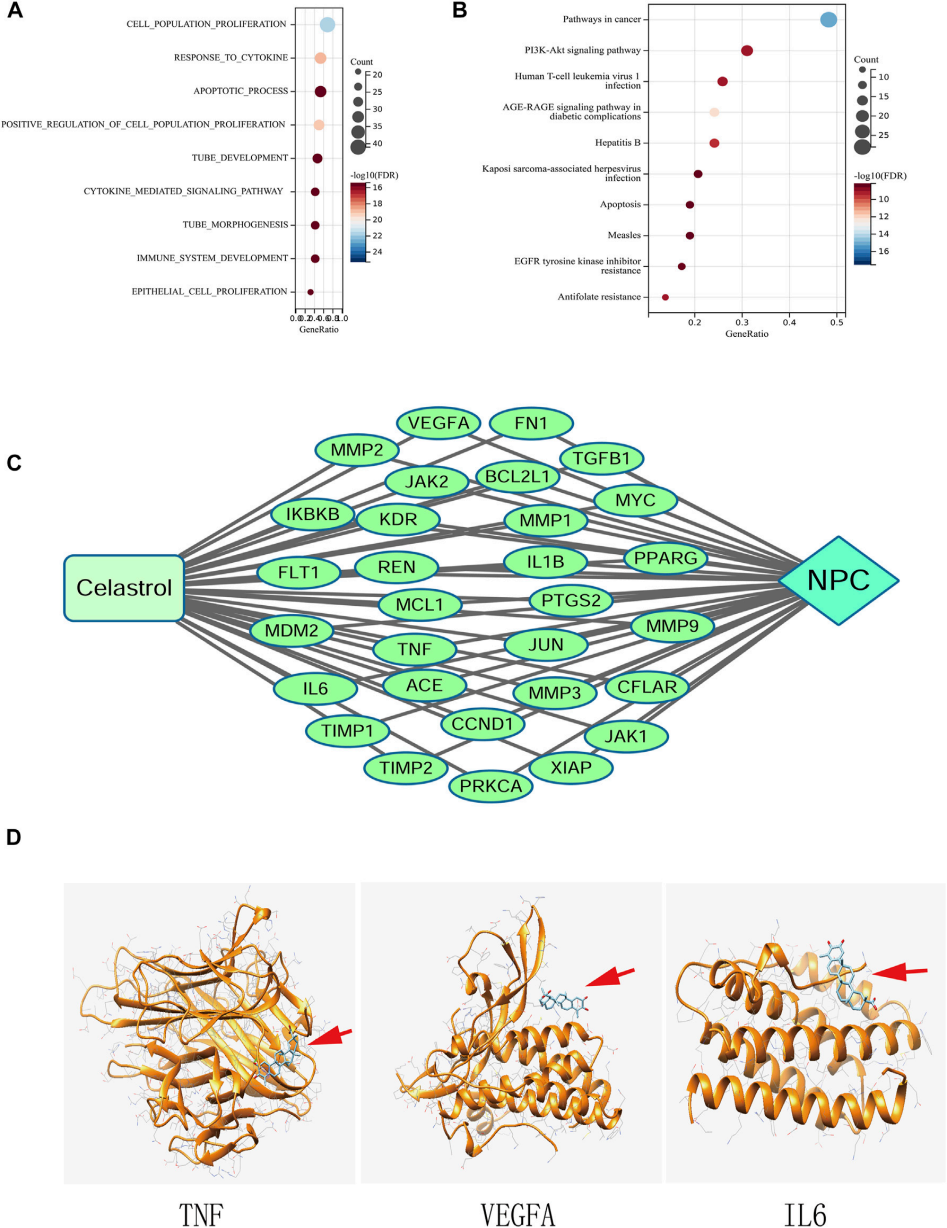
**(A)** GO analysis of the genes common to Celastrol and NPC. **(B)** KEGG pathway enrichment analysis. **(C)** The Compound-Target-Disease network. **(D)** Molecular docking between Celastrol and the top three hub genes (TNF, VEGFA, and IL6). The red arrows indicated the locations of Celastrol. The ribbons represented the 3D structures of the proteins.

The results of GO enrichment showed that these genes were enriched in more than 1000 GO entries by screening according to the standard of FDR <0.05. The top 10 GO terms are listed in Figure 3A. Based on these terms, genes are mainly enriched in four categories of functions as follows: 1) cell proliferation, such as cell population promotion, positive regulation of cell proliferation, and epithelial cell proliferation; 2) Apoptosis, such as apoptotic process; 3) immunity and cytokines, such as response to cytokines and cytokine-mediated signaling pathway; and 4) angiogenesis, such as tube development.

KEGG pathway enrichment results showed that these genes were enriched in 105 pathways. Figure 3B shows the top 10 signaling pathway entries, such as Pathways in cancer, PI3K-Akt signaling pathway, EGFR tyrosine kinase inhibitor resistance, Apoptosis, and AGE-RAGE signaling pathway in diabetic complications.

### Establishment of “compound-target-disease” network model

The interaction relationship among Celastrol, NPC, and gene targets was visualized using the Cytoscape software. Figure 3C shows the network model.

### Molecular docking prediction

The docking of Celastrol with the top three hub proteins, namely, TNF, VEGFA, and IL6 was shown in Figure 3D, in which Δ*G* (kcal/mol) less than 0 indicates that the molecules could be docked, and a score close to −7 indicated that the docking was good. The docking energy of TNF was −6.08, that of VEGFA was −6.76, and IL6 was −6.91.

### Validation of the candidate key genes *in vitro*

**FIGURE 4:**
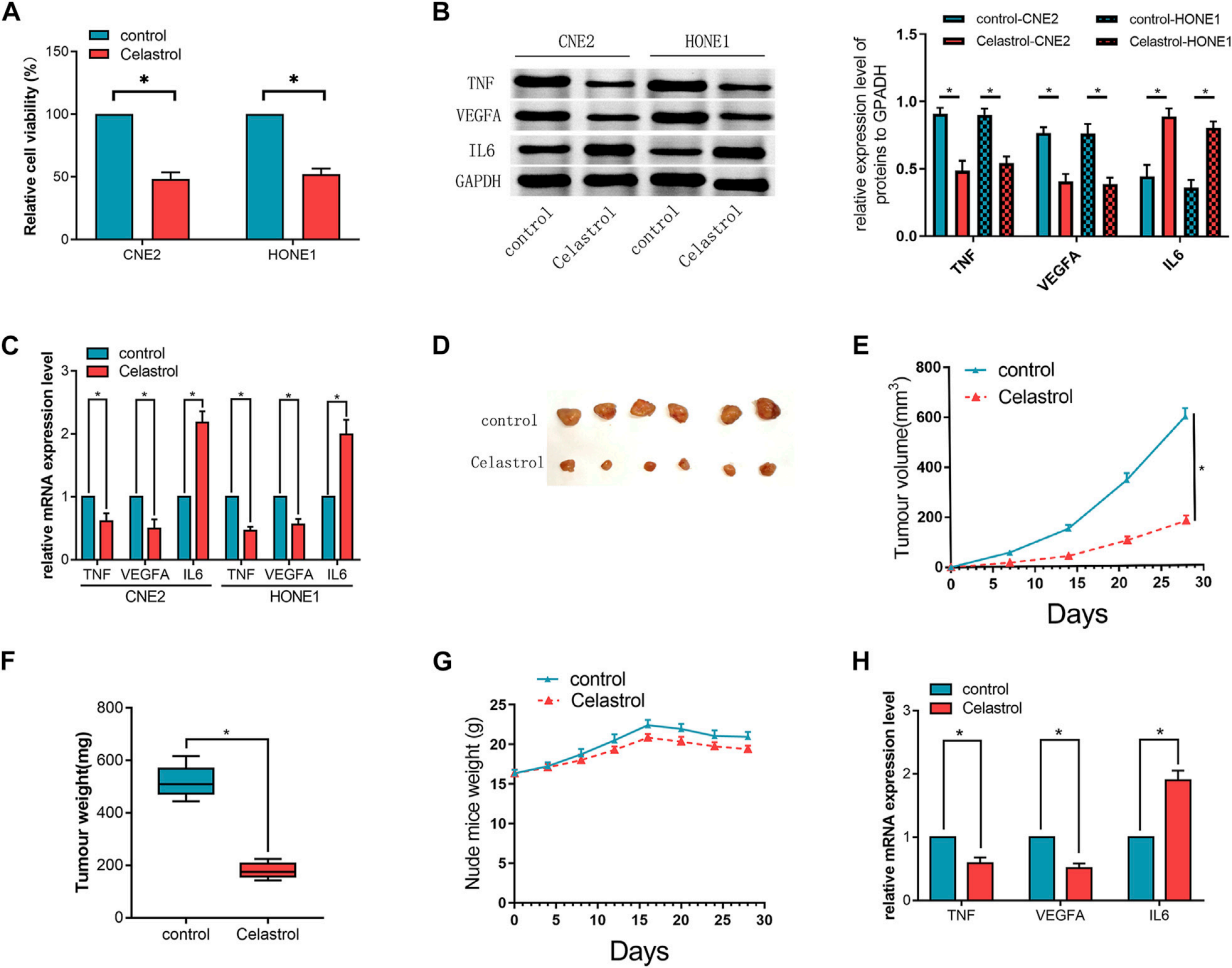
**(A)** The cell viability of the cells in the Celastrol-treated group was significantly lower than that of the cells in the control group. **(B,C)** The mRNA and protein expression levels of the candidate hub genes (TNF, VEGFA, and IL6) assessed by qRT-PCR **(C)** and western blot **(B)**, respectively, in CNE2 and HONE1 cells. **(D)** The excised transplanted tumors in two groups of mouse models. **(E)** The volumes of the transplanted tumors in the mice treated with or without Celastrol. **(F)** The weights of the tumors in the two groups. **(G)** The weight of the mice in the two groups. **(H)** The mRNA expression levels of the candidate hub genes (TNF, VEGFA, and IL6) in the transplanted tumors assessed by qRT-PCR.The *t*-test was used to compare between the two groups. (*n* = 3,*p < 0.05). The error bars represented the standard deviations.

NPC cells were treated with or without Celastrol for 24 h. The cell viability of the cells in the Celastrol-treated group was significantly lower than that of the cells in the control group (*p* < 0.05, Figure 4A). In the Celastrol-treated group, the mRNA and protein expressions of TNF and VEGFA were significantly decreased (*p* < 0.05) while the expression of IL-6 was increased relative to the controls, respectively (*p* < 0.05, Figures 4B,C).

### Validation of the candidate key genes *in vivo*

Further *in vivo* experiments showed that both the volume and the weight of the transplanted tumors were smaller in the Celastrol-treated group than those in the control group (*p* < 0.05, Figures 4D–F), indicating that the administration of Celastrol could inhibit the growth of the transplanted tumor *in vivo*. Celastrol treatment did not markedly induce changes in body weight in mice (Figure 4G). Next, the candidate genes in the tumors were also detected. The results showed that the expression trend of these genes was substantially consistent with that *in vitro* (Figure 4H).

## Discussion

*T. wilfordii* is a natural plant as well as a traditional Chinese medicine that has been widely used for the treatment of various diseases, such as nephritic syndrome, inflammatory bowel disease, systemic lupus erythematosus, rheumatoid arthritis, and other diseases (Song et al., 2020; Zhang Y et al., 2021; Tong et al., 2022). Celastrol is one of the important active substances extracted from *T. wilfordii*, and its antitumor activity has gradually attracted people’s attention (Lei et al., 2022). This study used *in silico* analysis to understand the targets and signaling pathways of Celastrol on NPC from multiple angles and levels. Sixty common targets were screened out, which are mainly enriched in multiple cell functions, such as cell proliferation, apoptosis, cytokines, and tube development. These genes may also participate in multiple signaling pathways, such as PI3K/Akt, EGFR tyrosine kinase inhibitor resistance, apoptosis, and AGE-RAGE signaling pathway in diabetic complications. Through the construction of a protein−protein interaction network, key genes such as TNF, VEGFA, and IL6 were identified.

Celastrol may exert anticancer activity through various mechanisms. For example, in pancreatic cancer cells, Celastrol plays a cytotoxic role by regulating various gene expressions, mainly through ATF3/DDIT3 overexpression and RRM2/MCM4 downregulation (Youns et al., 2021). Celastrol may inhibit the proliferation of cisplatin-resistant gastric cancer cells, induce their apoptosis, and reduce the expression of drug-resistant genes by inhibiting the expression of proteins related to the mTOR signal pathway (Zhan et al., 2021). Celastrol can reduce the activation of Notch-1 and the expression of HES-1 and HEY-1 of the downstream target protein and inhibit the stem cells of triple-negative breast cancer, thereby reducing the possibility of distant metastasis (Ramamoorthy et al., 2021). It can be seen that there are many molecular mechanisms involved in the inhibitory effect of Celastrol on tumors. In NPC, Celastrol can arrest the cell cycle of tumor cells in the G2/M phase and induce the activation of the apoptosis pathway, which may be mediated by the MAPK signaling pathway (Hsieh et al., 2019). In the process of inducing apoptosis of NPC cells, both the death receptor and mitochondrial apoptosis pathway are involved in this process (Lin et al., 2017). Therefore, the mechanism of Celastrol’s anticancer activity in NPC is also very complex, which can also explain why the common target genes obtained in this study are enriched in different pathways. However, limited studies have focused on the use of Celastrol for the treatment of NPC, and further studies are needed to verify the role of these pathways.

Among the common targets, some genes such as TNF, VEGFA, IL6, Myc, and CCND1 are in a pivotal position. These genes play a very important role in the occurrence and development of tumors. For example, in glioma, some non-coding RNAs promote tumor cell growth and tumor angiogenesis by regulating VEGFA expression (Zhang D et al., 2021). Moreover, for patients with ovarian cancer, VEGFA in serum is expected to replace CA125 and become a molecular marker for the early diagnosis of ovarian cancer (Maryam et al., 2021). Since M2 macrophages play an important role in tumor development and are associated with poor clinical outcomes (Li et al., 2022), TNFa may promote the development of NPC by recruiting M2 macrophage infiltration (Mardhiyah et al., 2021). IL6 is a cytokine that can interfere with the occurrence and development of tumors by activating downstream signal pathways. In NPC, IL6 activates JAK2/STAT3 signaling pathway to promote tumor progression, thus affecting the prognosis of patients (Zhuang et al., 2021). CCND1 overexpression was observed in a great proportion of clinical metastatic NPC tumors and was associated with poor outcomes. Thus, it also might be regarded as a target for NPC treatment (Hsu et al., 2018). In tumors, the low expression and short half-life of Myc can be reversed, which can lead to tumorigenesis. Proteins that directly interact with different Myc domains can affect the transcription of Myc target genes and regulate the stability of Myc through post-translational modifications, such as acetylation, methylation, and phosphorylation (Zhou et al., 2021). In acute myeloid leukemia, Myc directly inhibits the expression of TFEB (a transcription factor regulated by mTORC1) and regulates DNA methylation and cell differentiation (Wu and Eisenman, 2021). Therefore, these hub genes play a very important role in tumorigenesis and development, and Celastrol may inhibit NPC by interfering with the expression of these genes.

We examined the changes in TNF, VEGFA, and IL6 proteins after Celastrol treatment. Several studies have found that VEGFA is highly expressed in NPC cells and is associated with cell proliferation, invasion and metastasis, chemosensitivity, radiosensitivity, and other phenotypes of cancer cells (Chen et al., 2019; Chen et al., 2020; Guan et al., 2021). High expression of TNF is associated with macrophage polarization and may be an independent prognostic factor for NPC (Yu et al., 2019; Mardhiyah et al., 2021). Down-regulation of VEGFA and TNF expression was observed after Celastrol treatment and was accompanied by a decrease in cell viability, suggesting that Celastrol may reverse the malignant phenotype of NPC cells by reducing their expressions. Notably, IL6 is generally upregulated in NPC cells and its high expression is associated with cisplatin resistance and radioresistance (Yuan et al., 2021; Zhu et al., 2021), and nevertheless, Celastrol upregulated its expression. The discrepancy might be due to complex reasons. Cytokines of the IL6 family play an important role in mediating the tumor microenvironment to promote tumor inflammation and often act as diagnostic or prognostic biomarkers of tumors (Abaurrea et al., 2021). Although high IL6 expression is thought to have a tumor-promoting effect in many tumors, IL6 blockade therapy may exacerbate tumor growth because high IL6 expression increases the expression of cytokine signaling 3, which is required for the maintenance of antitumor M1 macrophage function (Beyranvand Nejad et al., 2021). In a study on melanoma, IL6-overexpressing tumors were found to grow significantly slower in mice with concomitant CD8^+^ T cell activation. Instead of observing a tumor-suppressive effect, accelerated tumor progression was monitored in melanoma mice treated with IL6-blocking antibodies (Weber et al., 2020). The evidence poses a challenge for tumor-targeted therapeutic strategies targeting IL6. This may help explain why Celastrol exerts an antitumor effect while increasing IL6 expression. However, future investigations are warranted to address this issue.

Although computer-simulated molecular docking technology suggests that there may be a direct binding relationship between Celastrol and the candidate proteins, the direct relationship needs further confirmation by experiments using specific methods, such as fluorescence polarization immunoassay (Yang et al., 2021), isothermal titration calorimetry (Kirley and Norman, 2022), and surface plasmon resonance (Campos de Paula et al., 2022). Evidence of direct interaction between the proteins and Celastrol is very helpful in providing insight into the mechanism of action of Celastrol. Of course, Celastrol may also regulate the target genes through indirect means, such as regulation between genes, and regulation between non-coding RNAs and genes. However, these validation steps are not involved in this study, which is expected to be carried out in future experiments.

DL can reflect whether a compound has the physical, chemical, and biological characteristics of becoming a drug, which is related to clinical efficacy (Wang et al., 2020). The greater the DL value, the safer the compound has absorption and metabolism, and the more likely it is to become a drug. “DL greater than 0.18” is often used as a safety index for drug screening. The DL value of Celastrol is 0.78, indicating that it has great potential to become a clinical drug. The Caco-2 cell model is a model that simulates the intestinal absorption of oral drugs. It is one of the classic models to study drug absorption and metabolism (Angelis and Turco, 2011). Caco-2 ≥ −0.4 is the standard for screening bioactive components, and the Caco-2 value of Celastrol is 0.46, suggesting that it can be fully absorbed by the small intestine. OB is one of the important pharmacokinetic parameters and an index to evaluate the absorption of drugs into the blood after entering the human body (Ding et al., 2022). Compounds with OB ≥ 30 have acceptable oral bioavailability. The OB value of Celastrol is 17.84, which is less than 30, suggesting that the oral bioavailability of Celastrol is poor, which may be related to the poor water solubility of Celastrol. To overcome this problem, nanoparticles have been used to increase the water solubility and oral bioavailability of Celastrol while reducing its toxicity to organs, and some results have been achieved (Wagh et al., 2021). However, the clinical application needs to ensure sufficient safety and effectiveness, and a large number of experiments need to be carried out in this regard.

This study has some limitations. First, in exploring the targets of Celastrol, not all commonly used databases can be searched for relevant drugs and targets, and different databases contain different targets because of different algorithms. Second, in the animal experimental part, some serological indicators can be considered, and also, the damage of Celastrol on solid organs such as the liver and kidney can be assessed. Third, considering the complexity of biological processes, the target genes obtained in this study are not necessarily the main genes of Celastrol acting in NPC. More experimental results, literature mining, or algorithm updates may reveal more gene targets and help us understand the molecular mechanism of Celastrol in the treatment of NPC more comprehensively. Therefore, the results of this study should be interpreted carefully. Fourth, in the *in vivo* experimental part, only one type of cell was used for transplantation tumor experiments. In future experiments, more experiments need to be performed with other cell lines to eliminate the bias of the results brought by the use of a single cell. In addition, whether the core genes have reciprocal relationships with each other needs to be investigated in future studies. However, the method and process of network pharmacology analysis used in this study are relatively mature, and the results obtained by this method are of great relevance as they point the way and provide clues for subsequent basic experiments. It is also important to note that female mice were used for animal experiments in this study, and although the experimental animals were not deliberately selected in terms of sex, this may have biased the experimental results in some ways. Moreover, the sample size of the experimental animals was only referred to other literature (Li et al., 2018; Xu et al., 2021) without detailed calculation. Therefore these issues need to be taken into account in future studies.

## Conclusion

This study uses bioinformatics methods to analyze the potential molecular mechanism of Celastrol for the treatment of NPC from multiple angles. The results suggest that Celastrol may play an intervention role in NPC through multiple targets, pathways, and angles. The results of this study will provide an important reference for subsequent basic and drug experiments. However, further experiments and repeated verification are needed to provide more information and thus uncover the anticancer mechanism of Celastrol.

## Data Availability

The datasets presented in this study can be found in online repositories. The names of the repository/repositories and accession number(s) can be found in the article/supplementary material.

## References

[B1] Abaurrea A. Araujo A. M. Caffarel M. M. (2021). The role of the IL-6 cytokine family in epithelial-mesenchymal plasticity in cancer progression. Int. J. Mol. Sci. 22 (15), 8334. 10.3390/ijms22158334 34361105 PMC8347315

[B2] Angelis I. D. Turco L. (2011). Caco-2 cells as a model for intestinal absorption. Curr. Protoc. Toxicol. Chapter 20, Unit20.6. 10.1002/0471140856.tx2006s47 21400683

[B3] Bai X. Fu R. J. Zhang S. Yue S. J. Chen Y. Y. Xu D. Q. (2021). Potential medicinal value of celastrol and its synthesized analogues for central nervous system diseases. Biomed. Pharmacother. 139, 111551. 10.1016/j.biopha.2021.111551 33865016

[B4] Beyranvand Nejad E. Labrie C. van Elsas M. J. Kleinovink J. W. Mittrucker H. W. Franken K. (2021). IL-6 signaling in macrophages is required for immunotherapy-driven regression of tumors. J. Immunother. Cancer 9 (4), e002460. 10.1136/jitc-2021-002460 33879600 PMC8061866

[B5] Bitencourt-Ferreira G. de Azevedo W. F. Jr. (2019). Docking with SwissDock. Methods Mol. Biol. 2053, 189–202. 10.1007/978-1-4939-9752-7_12 31452106

[B6] Cai Z. Qian B. Pang J. Tan Z. B. Zhao K. Lei T. (2022). Celastrol induces apoptosis and autophagy via the AKT/mTOR signaling pathway in the pituitary ACTH-secreting adenoma cells. Curr. Med. Sci. 42 (2), 387–396. 10.1007/s11596-022-2568-6 35419676

[B7] Campos de Paula H. M. Coelho Y. L. Benhame de Castro A. S. Marques I. A. Hudson E. A. de Paula Rezende J. (2022). Dynamics and energetics of bovine lactoferrin and phenylmethane dyes interaction followed by surface plasmon resonance. Colloids Surf. B Biointerfaces 219, 112794. 10.1016/j.colsurfb.2022.112794 36162180

[B8] Chen J. Lu F. Hu C. (2019). MicroRNA-299 targets VEGFA and inhibits the growth, chemosensitivity and invasion of human nasopharyngeal carcinoma cells. J. BUON 24 (5), 2049–2055. 31786874

[B9] Chen L. Lin G. Chen K. Wan F. Liang R. Sun Y. (2020). VEGF knockdown enhances radiosensitivity of nasopharyngeal carcinoma by inhibiting autophagy through the activation of mTOR pathway. Sci. Rep. 10 (1), 16328. 10.1038/s41598-020-73310-x 33004943 PMC7531011

[B10] Ding Q. Chen K. Liu X. Ding C. Zhao Y. Sun S. (2022). Modification of taxifolin particles with an enteric coating material promotes repair of acute liver injury in mice through modulation of inflammation and autophagy signaling pathway. Biomed. Pharmacother. 152, 113242. 10.1016/j.biopha.2022.113242 35691160

[B11] Guan X. Yu D. HuangFu M. Huang Z. Dou T. Liu Y. (2021). Curcumol inhibits EBV-positive Nasopharyngeal carcinoma migration and invasion by targeting nucleolin. Biochem. Pharmacol. 192, 114742. 10.1016/j.bcp.2021.114742 34428442

[B12] Hsieh M. J. Wang C. W. Lin J. T. Chuang Y. C. Hsi Y. T. Lo Y. S. (2019). Celastrol, a plant-derived triterpene, induces cisplatin-resistance nasopharyngeal carcinoma cancer cell apoptosis though ERK1/2 and p38 MAPK signaling pathway. Phytomedicine 58, 152805. 10.1016/j.phymed.2018.12.028 31022663

[B13] Hsu C. L. Lui K. W. Chi L. M. Kuo Y. C. Chao Y. K. Yeh C. N. (2018). Integrated genomic analyses in PDX model reveal a cyclin-dependent kinase inhibitor Palbociclib as a novel candidate drug for nasopharyngeal carcinoma. J. Exp. Clin. Cancer Res. 37 (1), 233. 10.1186/s13046-018-0873-5 30236142 PMC6149192

[B14] Hu W. J. Liu J. Zhong L. K. Wang J. (2018). Apigenin enhances the antitumor effects of cetuximab in nasopharyngeal carcinoma by inhibiting EGFR signaling. Biomed. Pharmacother. 102, 681–688. 10.1016/j.biopha.2018.03.111 29604587

[B15] Huang D. N. Wang S. Sooranna S. R. Miao J. H. (2021). The efficacy of natural bioactive compounds for the treatment of nasopharyngeal carcinoma. Mini Rev. Med. Chem. 21 (13), 1679–1691. 10.2174/1389557521666210105113831 33402084

[B16] Jin G. J. Peng X. Chen Z. G. Wang Y. L. Liao W. J. (2022). Celastrol attenuates chronic constrictive injury-induced neuropathic pain and inhibits the TLR4/NF-κB signaling pathway in the spinal cord. J. Nat. Med. 76 (1), 268–275. 10.1007/s11418-021-01564-4 34510370

[B17] Khalid Danish M. Gleeson J. P. Brayden D. J. Byrne H. J. Frias J. M. Ryan S. M. (2022). Formulation, characterisation and evaluation of the antihypertensive peptides, isoleucine-proline-proline and leucine-lysine-proline in chitosan nanoparticles coated with zein for oral drug delivery. Int. J. Mol. Sci. 23 (19), 11160. 10.3390/ijms231911160 36232463 PMC9570432

[B18] Kirley T. L. Norman A. B. (2022). Isothermal titration calorimetry determination of thermodynamics of binding of cocaine and its metabolites to humanized h2E2 anti-cocaine mAb. Biochem. Biophys. Rep. 32, 101354. 10.1016/j.bbrep.2022.101354 36186732 PMC9516381

[B19] Kouranov A. Xie L. de la Cruz J. Chen L. Westbrook J. Bourne P. E. (2006). The RCSB PDB information portal for structural genomics. Nucleic Acids Res. 34, D302–D305. 10.1093/nar/gkj120 16381872 PMC1347482

[B20] Lei Z. C. Li N. Yu N. R. Ju W. Sun X. N. Zhang X. L. (2022). Design and synthesis of novel celastrol derivatives as potential anticancer agents against gastric cancer cells. J. Nat. Prod. 85 (5), 1282–1293. 10.1021/acs.jnatprod.1c01236 35536757

[B21] Li M. He L. Zhu J. Zhang P. Liang S. (2022). Targeting tumor-associated macrophages for cancer treatment. Cell Biosci. 12 (1), 85. 10.1186/s13578-022-00823-5 35672862 PMC9172100

[B22] Li X. Zhu G. Yao X. Wang N. Hu R. Kong Q. (2018). Celastrol induces ubiquitin-dependent degradation of mTOR in breast cancer cells. Onco. Targets. Ther. 11, 8977–8985. 10.2147/OTT.S187315 30588010 PMC6294079

[B23] Lim H. Y. Ong P. S. Wang L. Goel A. Ding L. Li-Ann Wong A. (2021). Celastrol in cancer therapy: Recent developments, challenges and prospects. Cancer Lett. 521, 252–267. 10.1016/j.canlet.2021.08.030 34508794

[B24] Lin H. F. Hsieh M. J. Hsi Y. T. Lo Y. S. Chuang Y. C. Chen M. K. (2017). Celastrol-induced apoptosis in human nasopharyngeal carcinoma is associated with the activation of the death receptor and the mitochondrial pathway. Oncol. Lett. 14 (2), 1683–1690. 10.3892/ol.2017.6346 28789395 PMC5529953

[B25] Lin Y. Chen X. J. He L. Yan X. L. Li Q. R. Zhang X. (2022). Systematic elucidation of the bioactive alkaloids and potential mechanism from Sophora flavescens for the treatment of eczema via network pharmacology. J. Ethnopharmacol. 301, 115799. 10.1016/j.jep.2022.115799 36216196

[B26] Ling J. Wang Y. Ma L. Chang A. Meng L. Zhang L. (2022). Exploration of potential targets and mechanisms of fisetin in the treatment of non-small-cell lung carcinoma via network pharmacology and *in vitro* validation. Evid. Based. Complement. Altern. Med. 2022, 2383527. 10.1155/2022/2383527 PMC9208940 35733630

[B27] Liu Y. T. Chuang Y. C. Lo Y. S. Lin C. C. Hsi Y. T. Hsieh M. J. (2020). Asiatic acid, extracted from *Centella asiatica* and induces apoptosis pathway through the phosphorylation p38 mitogen-activated protein kinase in cisplatin-resistant nasopharyngeal carcinoma cells. Biomolecules 10 (2), E184. 10.3390/biom10020184 PMC7072674 31991751

[B28] Liu Z. Guo F. Wang Y. Li C. Zhang X. Li H. (2016). BATMAN-TCM: a bioinformatics analysis tool for molecular mechANism of traditional Chinese medicine. Sci. Rep. 6, 21146. 10.1038/srep21146 26879404 PMC4754750

[B29] Mardhiyah I. Ardiyan Y. N. Aliyah S. H. Sitepu E. C. Herdini C. Dwianingsih E. K. (2021). Necrosis factor-alpha (TNF-alpha) and the presence of macrophage M2 and T regulatory cells in nasopharyngeal carcinoma. Asian pac. J. Cancer Prev. 22 (8), 2363–2370. 10.31557/APJCP.2021.22.8.2363 34452547 PMC8629461

[B30] Maryam N. Ahmed S. S. Alam R. Hanif M. U. Saleem M. Gul R. (2021). Role of serum VEGF-A biomarker for early diagnosis of ovarian cancer instead of CA-125. J. Pak. Med. Assoc. 71 (9), 2192–2197. 10.47391/JPMA.05-688 34580513

[B31] Mei S. (2018). *In silico* enhancing *M. tuberculosis* protein interaction networks in STRING to predict drug-resistance pathways and pharmacological risks. J. Proteome Res. 17 (5), 1749–1760. 10.1021/acs.jproteome.7b00702 29611419

[B32] Momtazi-Borojeni A. A. Ghasemi F. Hesari A. Majeed M. Caraglia M. Sahebkar A. (2018). Anti-cancer and radio-sensitizing effects of Curcumin in nasopharyngeal carcinoma. Curr. Pharm. Des. 24 (19), 2121–2128. 10.2174/1381612824666180522105202 29788875

[B33] Noor F. Tahir Ul Qamar M. Ashfaq U. A. Albutti A. Alwashmi A. S. S. Aljasir M. A. (2022). Network pharmacology approach for medicinal plants: Review and assessment. Pharm. (Basel) 15 (5), 572. 10.3390/ph15050572 PMC9143318 35631398

[B34] Peng H. Shahidi F. (2022). The effects of acyl chain length on antioxidant efficacy of mono- and multi-acylated resveratrol: A comparative assessment. Molecules 27 (3), 1001. 10.3390/molecules27031001 35164266 PMC8839368

[B35] Peng X. Zhou Y. Tao Y. Liu S. (2021). Nasopharyngeal carcinoma: The role of the EGFR in epstein-barr virus infection. Pathogens 10 (9), 1113. 10.3390/pathogens10091113 34578147 PMC8470510

[B36] Ramamoorthy P. Dandawate P. Jensen R. A. Anant S. (2021). Celastrol and triptolide suppress stemness in triple negative breast cancer: Notch as a therapeutic target for stem cells. Biomedicines 9 (5), 482. 10.3390/biomedicines9050482 33924995 PMC8146582

[B37] Ru J. Li P. Wang J. Zhou W. Li B. Huang C. (2014). TCMSP: a database of systems pharmacology for drug discovery from herbal medicines. J. Cheminform. 6, 13. 10.1186/1758-2946-6-13 24735618 PMC4001360

[B38] Song C. Y. Xu Y. G. Lu Y. Q. (2020). Use of Tripterygium wilfordii hook F for immune-mediated inflammatory diseases: progress and future prospects. J. Zhejiang Univ. Sci. B 21 (4), 280–290. 10.1631/jzus.B1900607 32253838 PMC7183448

[B39] Stelzer G. Dalah I. Stein T. I. Satanower Y. Rosen N. Nativ N. (2011). *In-silico* human genomics with GeneCards. Hum. Genomics 5 (6), 709–717. 10.1186/1479-7364-5-6-709 22155609 PMC3525253

[B40] Su Z. Y. Siak P. Y. Leong C. O. Cheah S. C. (2022). Nasopharyngeal carcinoma and its microenvironment: Past, current, and future perspectives. Front. Oncol. 12, 840467. 10.3389/fonc.2022.840467 35311066 PMC8924466

[B41] Tong X. Qiao Y. Yang Y. Liu H. Cao Z. Yang B. (2022). Applications and mechanisms of Tripterygium wilfordii hook. F. and its preparations in kidney diseases. Front. Pharmacol. 13, 846746. 10.3389/fphar.2022.846746 35387327 PMC8977547

[B42] Wagh P. R. Desai P. Prabhu S. Wang J. (2021). Nanotechnology-based celastrol formulations and their therapeutic applications. Front. Pharmacol. 12, 673209. 10.3389/fphar.2021.673209 34177584 PMC8226115

[B43] Wang K. X. Gao Y. Gong W. X. Ye X. F. Fan L. Y. Wang C. (2020). A novel strategy for decoding and validating the combination principles of huanglian jiedu decoction from multi-scale perspective. Front. Pharmacol. 11, 567088. 10.3389/fphar.2020.567088 33424585 PMC7789881

[B44] Wang P. Wang S. Chen H. Deng X. Zhang L. Xu H. (2021). TCMIP v2.0 powers the identification of chemical constituents available in xinglou chengqi decoction and the exploration of pharmacological mechanisms acting on stroke complicated with tanre fushi syndrome. Front. Pharmacol. 12, 598200. 10.3389/fphar.2021.598200 34335236 PMC8320350

[B45] Weber R. Riester Z. Huser L. Sticht C. Siebenmorgen A. Groth C. (2020). IL-6 regulates CCR5 expression and immunosuppressive capacity of MDSC in murine melanoma. J. Immunother. Cancer 8 (2), e000949. 10.1136/jitc-2020-000949 32788238 PMC7422659

[B46] Wu X. Eisenman R. N. (2021). MYC and TFEB control DNA methylation and differentiation in AML. Blood Cancer Discov. 2 (2), 116–118. 10.1158/2643-3230.Bcd-20-0230 34661153 PMC8447267

[B47] Xu Q. Chen G. Xu H. Xia G. Zhu M. Zhan H. (2021). Celastrol attenuates RANKL-induced osteoclastogenesis *in vitro* and reduces titanium particle-induced osteolysis and ovariectomy-induced bone loss *in vivo* . Front. Pharmacol. 12, 682541. 10.3389/fphar.2021.682541 34149427 PMC8210420

[B48] Yang H. He Q. Eremin S. A. Pan J. Zou Y. Cui X. (2021). Fluorescence polarization immunoassay for rapid determination of dehydroepiandrosterone in human urine. Anal. Bioanal. Chem. 413 (17), 4459–4469. 10.1007/s00216-021-03403-7 34137913

[B49] Yao S. S. Han L. Tian Z. B. Yu Y. N. Zhang Q. Li X. Y. (2019). Celastrol inhibits growth and metastasis of human gastric cancer cell MKN45 by down-regulating microRNA-21. Phytother. Res. 33 (6), 1706–1716. 10.1002/ptr.6359 30989726

[B50] Youns M. Askoura M. Abbas H. A. Attia G. H. Khayyat A. N. Goda R. M. (2021). Celastrol modulates multiple signaling pathways to inhibit proliferation of pancreatic cancer via DDIT3 and ATF3 up-regulation and RRM2 and MCM4 down-regulation. Onco. Targets. Ther. 14, 3849–3860. 10.2147/OTT.S313933 34194230 PMC8238076

[B51] Yu Y. Ke L. Xia W. X. Xiang Y. Lv X. Bu J. (2019). Elevated levels of TNF-alpha and decreased levels of CD68-positive macrophages in primary tumor tissues are unfavorable for the survival of patients with nasopharyngeal carcinoma. Technol. Cancer Res. Treat. 18, 1533033819874807. 10.1177/1533033819874807 31522611 PMC6747870

[B52] Yuan X. Zhang L. Huang Y. Liu D. Peng P. Liu S. (2021). Induction of interleukin-6 by irradiation and its role in epithelial mesenchymal transition and radioresistance of nasopharyngeal carcinoma cells. Head. Neck 43 (3), 757–767. 10.1002/hed.26531 33150659

[B53] Zhan D. Ni T. Wang H. Lv M. Sunagawa M. Liu Y. (2021). Celastrol inhibits the proliferation and decreases drug resistance of cisplatin-resistant gastric cancer SGC7901/DDP cells. Anticancer. Agents Med. Chem. 22, 270–279. 10.2174/1871520621666210528144006 34053427

[B54] Zhang D. Jiang H. Ye J. Gao M. Wang X. Lu E. (2021). A novel lncRNA, RPL34-AS1, promotes proliferation and angiogenesis in glioma by regulating VEGFA. J. Cancer 12 (20), 6189–6197. 10.7150/jca.59337 34539892 PMC8425216

[B55] Zhang Y. Mao X. Li W. Chen W. Wang X. Ma Z. (2021). Tripterygium wilfordii: An inspiring resource for rheumatoid arthritis treatment. Med. Res. Rev. 41 (3), 1337–1374. 10.1002/med.21762 33296090

[B56] Zhou Y. Gao X. Yuan M. Yang B. He Q. Cao J. (2021). Targeting Myc interacting proteins as a winding path in cancer therapy. Front. Pharmacol. 12, 748852. 10.3389/fphar.2021.748852 34658888 PMC8511624

[B57] Zhu L. Zhao J. Zhang Y. Zhou W. Yin L. Wang Y. (2018). ADME properties evaluation in drug discovery: in silico prediction of blood-brain partitioning. Mol. Divers. 22 (4), 979–990. 10.1007/s11030-018-9866-8 30083853

[B58] Zhu X. Liu L. Wang Y. Cong J. Lin Z. Wang Y. (2021). lncRNA MIAT/HMGB1 Axis is involved in cisplatin resistance via regulating IL6-mediated activation of the JAK2/STAT3 pathway in nasopharyngeal carcinoma. Front. Oncol. 11, 651693. 10.3389/fonc.2021.651693 34094941 PMC8173225

[B59] Zhuang M. Ding X. Song W. Chen H. Guan H. Yu Y. (2021). Correlation of IL-6 and JAK2/STAT3 signaling pathway with prognosis of nasopharyngeal carcinoma patients. Aging (Albany NY) 13 (12), 16667–16683. 10.18632/aging.203186 34165442 PMC8266356

